# Ultrasonic Monitoring of the Processes of Blast Freezing and Thawing of Meat ^†^

**DOI:** 10.3390/foods15020328

**Published:** 2026-01-16

**Authors:** Alexey Tatarinov, Marija Osipova, Viktors Mironovs

**Affiliations:** 1 Institute of Electronics and Computer Sciences, LV-1006 Riga, Latvia; 2 Faculty of Civil and Mechanical Engineering, Riga Technical University, LV-1048Riga, Latvia

**Keywords:** pork, freezing and thawing, ultrasonic testing, signal patterns, lean and fat tissue

## Abstract

Freezing and thawing affect the structural integrity and quality of meat, yet these processes remain difficult to monitor due to spatial temperature gradients and non-uniform phase transitions. This study investigates the ability of ultrasound to detect dynamic freezing and thawing events in pork tissues with different fat contents. Specimens of water, lean meat, marbled meat, layered lean–fat structures, and lard were subjected to controlled freeze–thaw cycles while ultrasonic signals and internal temperatures were continuously monitored. Consistent amplitude drops in the megahertz range at entering the freezing phase formed characteristic signal patterns that differed sharply between lean and fatty tissues. Lean meat, dominated by water content, exhibited rapid signal loss at the onset of ice crystallization and a clear recovery of amplitude once fully frozen. Fat-rich tissues demonstrated prolonged attenuation and near disappearance of high-frequency signals, with incomplete recovery even at deep-frozen states. A jump of ultrasound velocity from 1.4–1.6 km/s in a warm state to 2.6–3.7 km/s in a frozen state indicated complete freezing. Hysteresis between temperature readings and actual phase transition moments was found. Distinct ultrasonic freeze–thaw signatures reflecting tissue composition suggest a novel approach for monitoring the true onset and completion of freezing and thawing in meat.

## 1. Introduction

Freezing remains the cornerstone of meat preservation. However, conventional freezing is often accompanied by textural deterioration, drip loss, and nutritional degradation, prompting continuous development of advanced freezing and thawing technologies aimed at minimizing structural damage [1]. Among the established industrial solutions, blast freezing remains dominant due to its scalability, cost-effectiveness, and adaptability for large meat cuts and high-volume cold-chain logistics. Its ability to form relatively small ice crystals offers improved preservation of texture and reduced thaw drip compared with slow freezing. With the growing global demand for frozen foods and ongoing investment in cold-chain infrastructure, blast freezing continues to expand as a practical and reliable technology. To improve freezing uniformity and reduce quality loss during thawing, numerous advanced methods have been proposed, such as ultrasonic freezing, high-voltage electric and magnetic fields, liquid nitrogen, and high-pressure systems [2,3].

Despite these technological advances, in situ, real-time monitoring of freezing and thawing remains a critical challenge. Freezing is inherently non-uniform, as internal temperature gradients lead to heterogeneous ice crystal formation, affecting structural integrity, drip loss, and sensory attributes. Without internal monitoring, operators cannot detect partial freezing, incomplete thawing, or non-uniform cooling. Real-time sensing therefore plays a crucial role in verifying that regulatory temperature thresholds are met, preventing unnoticed temperature abuse, improving energy efficiency, and enabling automated freezer control systems and AI-based quality prediction models.

Several non-destructive sensing technologies have been explored for monitoring thermal and structural changes in meat, each offering unique advantages [4]. Ultrasound, operated in pulse-echo or through-transmission mode, is particularly promising because it responds sensitively to changes in acoustic impedance during phase transitions. Ultrasound can track ice front propagation, microstructural alterations, and thermal events in real time [5]. Recent studies demonstrate its ability to monitor pre-thawing stages with substantial amplitude changes [6], predict thawing endpoints through machine learning models [7], and detect ice crystal formation during super-chilling using combined ultrasound and near-infrared spectroscopy [8]. Complementary methods include low-field NMR (for water mobility), infrared thermography (for surface temperature mapping), and electrochemical sensors (protein oxidation indicators) [9,10,11].

Ultrasound holds several distinct advantages for industrial applications. Changes in acoustic velocity, attenuation, and backscatter correlate strongly with phase transitions, enabling highly sensitive detection of the nucleation events, freezing completeness, thawing progression, and internal structural changes that temperature sensors cannot capture [6]. Moreover, ultrasound penetrates several centimeters into tissue, operates reliably in cold and humid environments, and integrates well with machine learning algorithms for stage classification.

The purpose of this Communication is to present new experimental findings on the ultrasonic monitoring of meat during freezing and thawing, obtained from a limited set of specimens spanning a wide range of fat contents. We report unique ultrasonic pattern behaviors associated with varying fat composition, as well as measurable delays between the true physical freezing and thawing transitions and the moment when temperature sensors cross the 0 °C threshold. The results were obtained using a custom laboratory setup designed for rapid freezing of small meat specimens with integrated ultrasonic recording capabilities, enabling observation of dynamic freeze–thaw behavior [12].

## 2. Materials and Methods

### 2.1. Experimental Setup

**Figure 1:**
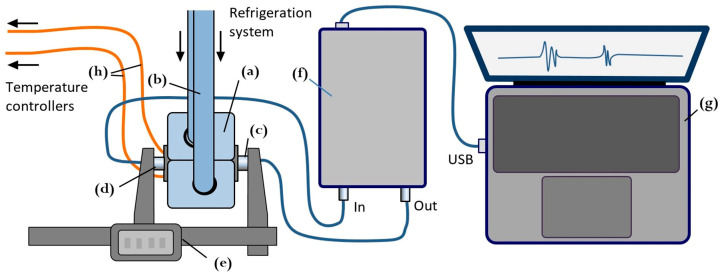
Diagram of ultrasonic measurement setup for monitoring freezing–defrosting process: (**a**) meat specimen enclosed in claws of refrigeration system; (**b**) refrigerant tubes; (**c**) ultrasonic emitter; (**d**) ultrasonic receiver; (**e**) digital caliper with transducers’ consoles; (**f**) ultrasonic acquisition unit; (**g**) laptop; and (**h**) thermocouples.

**Figure 2:**
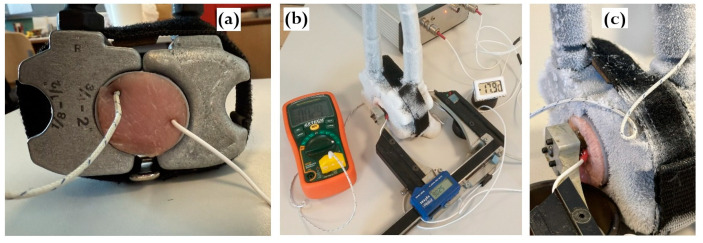
Illustrations of ultrasonic experiment. (**a**) Close-up view of meat specimen in refrigerator’s claws with inserted thermocouples; (**b**) measurement assembly; (**c**) close-up view of ultrasonic transducers attached to specimen during freezing.

Figure 1 shows the configuration of the ultrasonic measurement system used to record continuously acoustic signals throughout the freeze–thaw cycle. Meat specimens were placed inside cylindrical plastic capsules (Ø40 × 60 mm, wall thickness 1 mm) and rigidly mounted between the freezing claws of a refrigeration system. Internal temperature was measured using two calibrated thermocouples connected to DT890C+ multimeters (Zhangzhou Weihua Electronic Co., Zhangzhou, China). To account for spatial temperature gradients in the specimen, one thermocouple was positioned at the specimen’s periphery, and the other one at the core, the latter being inserted at an angle to avoid interference with ultrasound propagation (Figure 2a). Once installed, the acoustic base remained unchanged throughout all measurements during the freezing and thawing experiment (Figure 2b,c). Freezing was performed using a REMS Frigo 2 F-Zero electric pipe-freezing system (REMS Messtechnik GmbH & Co KG, Waiblingen, Germany) and operating with high-purity propane R-290 as the refrigerant [12]. The system supports low-volume freezing studies with power consumption below 500 W. Freezing began at +20 °C and continued until the central thermocouple reached −20 °C. Freezing rates ranged from 0.25 to 3 °C/min depending on temperature range and specimen heat capacity. The freezing rate was not constant through the freezing process, slowing down as the phase transition was passed. After reaching −20 °C, freezing was terminated and thawing was initiated by gently heating the freezing claws with a hot air gun.

### 2.2. Ultrasonic Measurements

Ultrasound measurements were taken at discrete temperature intervals during the freeze–thaw cycle in through transmission mode using a pair of 2.2 MHz piezoelectric transducers. The transducers with parallel surfaces were coaxially mounted on a caliper-based probe to ensure stable pressure and precise readings of the acoustic base. Signals were recorded using a custom acquisition system built on an Altera Cyclone IV FPGA (San Jose, CA, USA) with USB 3.0 FIFO transfer FT600Q (Glasgow, UK). The system transmitted 2-period tone bursts at a 2.2 MHz carrier frequency and a voltage of 140 Vpp, with a 10-bit ADC resolution, 30 MHz sampling, and 16-fold averaging. When changing and restoring acoustic contact on the same meat specimen at a constant room temperature, the system ensured reproducibility of ultrasound velocity measurements within ±2 m/s in the lean specimen and ±5 m/s in the fatty specimen. When measuring signal intensity, the reproducibility was ±10% and ±15%, respectively.

### 2.3. Specimens

**Figure 3:**
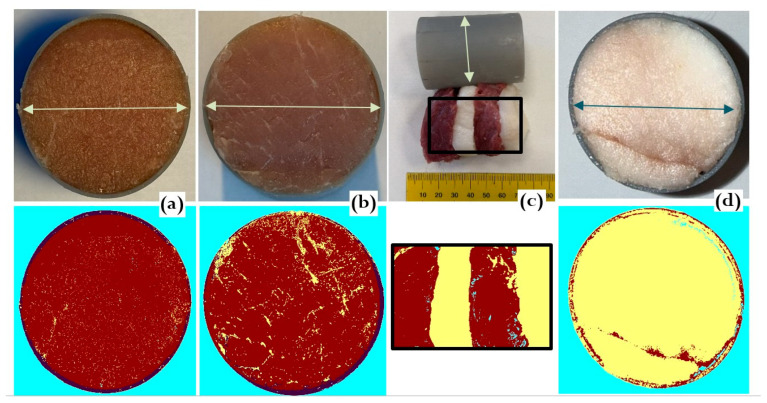
Meat specimens. (**a**) Lean pork; (**b**) marbled pork; (**c**) layered structure of lean meat and fat; (**d**) lard. Top row—original images; bottom row—segmented images, where lean tissue is marked in dark red and fat in yellow. Arrows indicate the capsule diameter of 40 mm.

The experiment included five specimens designed to represent a wide range of fat contents to reveal the corresponding ultrasonic trends in the freeze–thaw cycle (Figure 3):

1. Water (reference for freezing behavior of high water-content tissues). Lean pork contains 72–76% water [13];
2. Lean pork (<4% fat);
3. Marbled pork (~10% fat);
4. Layered lean–fat structure (alternating layers of lard and lean meat perpendicular to ultrasound propagation path; ~45% fat);
5. Lard. (~99% fat).

All specimens were prepared as 60 mm thick, 40 mm diameter cuts to be placed inside the corresponding plastic capsules. The water specimen was prepared in the same capsule, sealed inside a thin rubber membrane. Fat and lean proportions were estimated by RGB-based image segmentation using pattern recognition software in C#, calculating the number of pixels corresponding to the proximity to lean and fat classes.

At this exploratory stage of the pilot study, the non-automated and labor-intensive measurement protocol precluded systematic reproducibility testing and detailed analysis of influencing factors, with the primary objective to explore qualitatively trends in the evolution of acoustic parameters across tissues with increasing fat content.

## 3. Results and Discussion

### 3.1. Signal Behavior During Freezing

**Figure 4:**
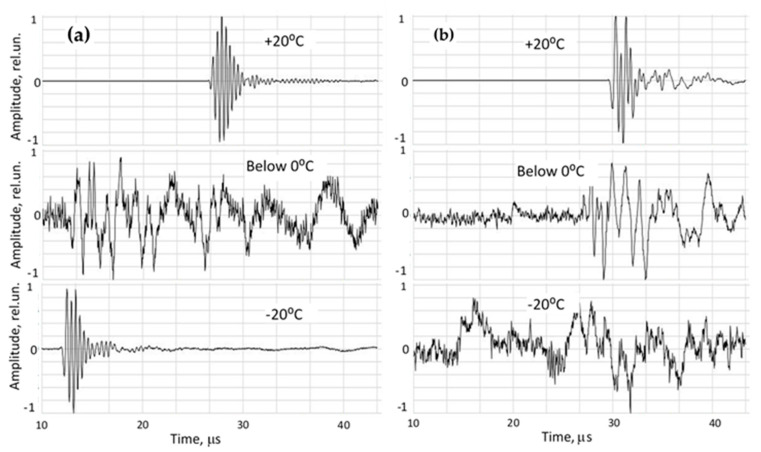
Changes in ultrasonic waveforms in (**a**) lean pork and in (**b**) lard during freezing.

Ultrasonic signal evaluation during cooling is illustrated in Figure 4 for lean pork and lard. Across all specimens, including water, signal amplitudes decreased markedly during the moment of phase transition. In warm tissue (above 0 °C), direct-propagation signals exhibited high signal-to-noise ratios (SNRs) in the range of 300–500. During freezing, amplitudes dropped to SNR values 3–8.

However, lean and fatty tissues displayed fundamentally different signal trajectories as temperature decreased to −20 °C.

(a) Lean meat (high water content), characterized by:
  - Rapid disappearance of signals indicated the coexistence of liquid water and ice, where emerging crystals scatter ultrasound and increase attenuation.
  - Once fully frozen, the specimen formed a more homogeneous acoustic medium, making it possible to partially recover the signal (SNR 50–100).
  - Ultrasound velocity increased sharply as propagation transitioned from liquid-like tissue to solid-like (ice) behavior.
(b) Fatty tissue (lard), characterized by:
  - Signal amplitude declined continuously across the full temperature range.
  - MHz-range signals disappeared completely at deep-frozen states.
  - Only weak low-frequency components persisted in frozen fat.

### 3.2. Stepwise Freeze–Thaw Profiles

**Figure 5:**
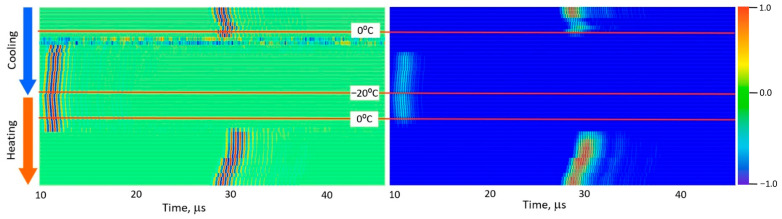
Diagrams of ultrasonic signal changes in water during freezing and thawing. One line corresponds to the ultrasonic signal on a time scale with color-coded amplitude normalized by peak from −1.0 to 1.0. The left diagram shows signals normalized by the peak amplitude within each signal; the right diagram shows rectified signals normalized by the peak amplitude across all signals. The freezing (cooling) process is indicated by a blue arrow and the thawing (heating) process by a red arrow. Transitions through a temperature of zero degrees and reaching −20 °C are marked by red horizontal lines.

**Figure 6:**
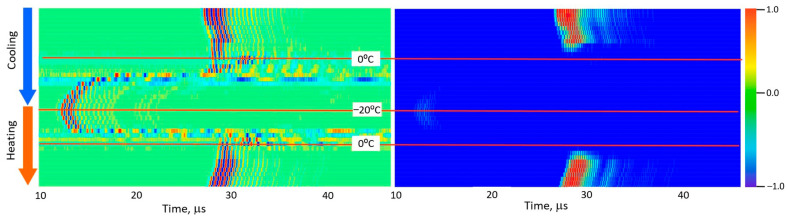
Diagrams of ultrasonic signal changes in lean pork meat during freezing and thawing. The designations are the same as in Figure 5.

**Figure 7:**
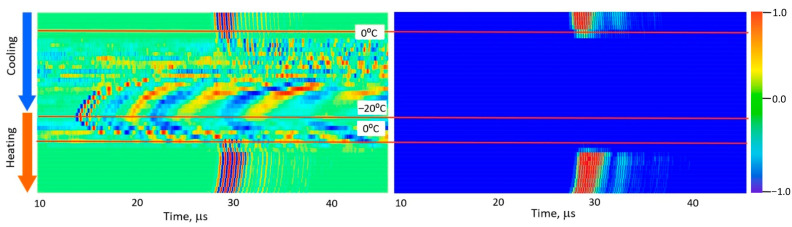
Diagrams of ultrasonic signal changes in marbled pork meat during freezing and thawing. The designations are the same as in Figure 5.

**Figure 8:**
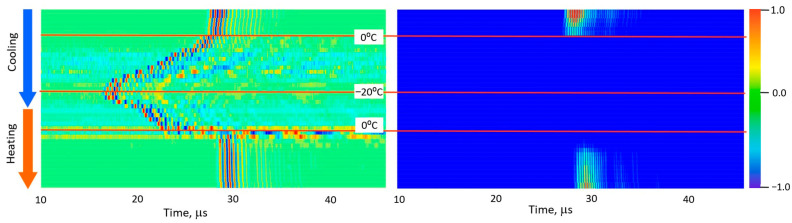
Diagrams of ultrasonic signal changes in layered lean/fat pork meat during freezing and thawing. The designations are the same as in Figure 5.

**Figure 9:**
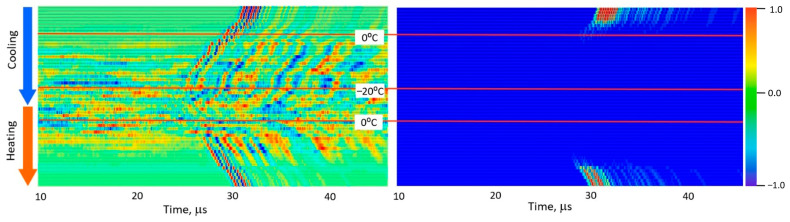
Diagrams of ultrasonic signal changes in pork lard during freezing and thawing. The designations are the same as in Figure 5.

Figure 5, Figure 6, Figure 7, Figure 8 and Figure 9 present normalized ultrasonic signals during gradual cooling/freezing and subsequent heating/thawing processes in a sequence of the specimens from water and minimal fat content in meat to high fat content and lard. Left-hand plots normalize each signal to its own peak amplitude (emphasizing velocity-related temporal shifts), while right-hand plots normalize all measurements to the global peak (highlighting amplitude attenuation).

Key observed trends:

(a) A strong amplitude drop at the onset of freezing in all tissues.
(b) Reappearance of MHz-range signals at deep-frozen states in lean meat, but not in fat-rich specimens.
(c) Faster freezing and thawing transitions in lean meat compared with fatty meat.
(d) Symmetrical acoustic behavior during cooling and heating, forming distinctive freeze–thaw “gates.”
(e) Pronounced hysteresis—actual freeze/thaw, where true phase transitions lag behind the temperature crossing 0 °C due to internal temperature gradients and thermal hysteresis in water, organic liquids, and fat [14,15].

These findings indicate that each tissue type produces a characteristic ultrasonic freeze–thaw signature, effectively serving as an acoustic fingerprint linked to fat content and internal structure.

### 3.3. Quantitative Analyses

**Figure 10:**
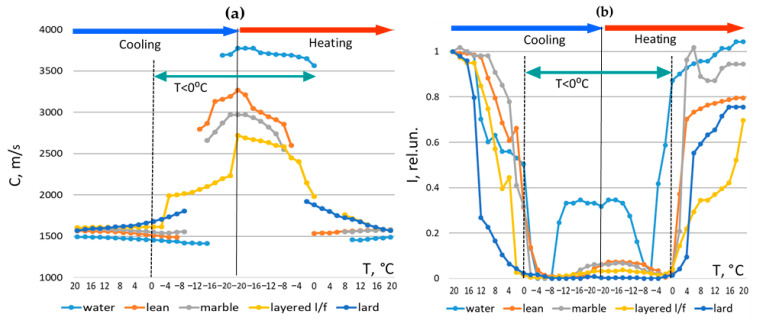
Changes in ultrasound velocity *C* (**a**) and ultrasonic signal intensity *I* (**b**) with temperature T change during freezing and thawing processes in water, fat (lard) and different types of meat.

Ultrasound velocity and signal intensity were extracted from recorded waveforms (Figure 10). Velocity was calculated using transmission time corrected for the transducers constant with the first crossing of zero degrees as the signal reference point and calibrated acoustic base. Signal intensity was obtained by integrating amplitudes after noise correction.

Results of the analysis of changes in the velocity and intensity parameters:

(a) Lean meat behaved similarly to water, exhibiting sharp velocity transitions and substantial intensity recovery after complete freezing.
(b) Fatty tissues exhibited behavior closer to pure fat, with minimal velocity increase and persistent intensity loss.
(c) A sharp intensity drop consistently marked the onset of freezing.
(d) A velocity jump from 1400–1600 m/s in a warm state to >2000 m/s in a frozen state indicated complete freezing for all types of meat.
(e) Restoration of velocity and intensity to near-initial values indicated complete thawing for all types of meat.
(f) Extremely low intensities and abrupt transitions corresponded to incomplete or partial freezing or thawing occurring due to temperature gradients and hysteresis effects.

At positive temperatures, when cooling from +20 to 0 degrees Celsius, the behavior of the velocity and intensity were consistent with classical observations [16]. Ultrasound velocity decreased upon cooling in non-fatty water-rich specimens but increased in the fat specimen. In the layered lean–fat specimen, these opposing trends overlapped. The intensity drop upon cooling was sharper for fatty specimens, confirming the increase in attenuation in fat with decreasing temperature.

The stronger attenuation observed in fatty tissues during cooling confirms the known fact that ultrasound attenuation is higher and ultrasound velocity is lower in adipose tissue than in muscle tissue [17,18]. Limited data exist on the acoustic behavior of fat during freezing. However, the various biophysical and biomolecular transformations that adipose tissue undergoes during freezing are due to the phase behavior of lipids and their interactions with water ice formation. When the temperature drops below the melting points of its constituent triglycerides and fatty acids, the lipid phase solidifies through polymorphic crystalline transitions, leading to changes in the microstructure and mechanical rigidity [19,20]. In the residual aqueous fractions, ice crystallization concentrates dissolved substances. Taken together, these processes can lead to increased attenuation during freezing and hysteresis effects.

### 3.4. Research Outlook

The results reveal a consistent qualitative trend observed across the sequence water → lean meat → marbled meat → fatty meat → lard, indicating the sensitivity of the ultrasonic response to tissue composition. Comprehensive quantitative and statistical analysis will be addressed in a future continued research, requiring systematic assessment of reproducibility and accounting for factors such as freezing and thawing rates, specimen size and mass, and meat type.

Future work should also aim to validate ultrasonic monitoring on larger industrial-scale cuts and systematically assess how process variables such as specimen size, cooling and heating rates, and meat type influence acoustic responses during freezing and thawing. Quality of frozen and thawed meat are known to be affected by such factors as cooling rate and product size significantly influencing ice crystal formation, which in turn governs microstructural damage, water-holding capacity, and sensory attributes such as color and juiciness [21].

Comprehensive reviews on meat freezing and thawing describe the wide range of physicochemical quality parameters that are altered by phase change dynamics, including moisture loss, protein denaturation, lipid oxidation, texture changes, and highlight how these depend on freezing and thawing conditions [22]. In this context, integrating ultrasonic sensing with automated freezer controls could provide real-time insight into internal phase transitions that correlate with known quality outcomes, complementing traditional temperature measurements. However, robust validation across different product geometries, freezing regimes, and species is needed before the approach can be broadly applied in industrial settings.

In addition, the ultrasound monitoring method is well suited for studying regimes of shock freezing with additional effects on meat, for example, when applying electromagnetic fields [23] or ultrasonic processing [24] simultaneously with cooling. The method is in line with modern trends in using ultrasound velocity and machine learning algorithms to monitor freezing processes [7].

## 4. Conclusions

Ultrasonic measurements captured distinct acoustic patterns during freezing and thawing across meat types with varying fat content. Lean meat exhibited rapid signal attenuation at the onset of ice formation and a clear recovery of amplitude upon complete freezing, whereas fatty tissues showed sustained attenuation and, in some cases, complete loss of MHz-range signals throughout deep freezing. All specimens demonstrated notable hysteresis between temperature readings and true physical transitions in freezing and thawing.

These preliminary findings suggest that ultrasound may serve as a non-destructive, real-time indicator of phase transitions during blast freezing and thawing of meat. Differences in acoustic patterns observed between lean and fatty specimens indicate potential sensitivity to tissue composition and freezing behavior. Although exploratory in nature, the results support the feasibility of ultrasonic monitoring of internal freezing and thawing dynamics and provide motivation for further investigations involving larger sample sets to assess its relevance for process monitoring and quality evaluation in industrial meat freezing.

Further studies should aim to validate the method on larger, industrial-scale cuts, to account for other factors influencing freezing and thawing such as the object’s size, cooling/heating rate, and types of meat, as well as to integrate ultrasonic sensing with automated freezer controls. Expanding frequency ranges and developing robust industrial transducer mounts will enhance applicability in real processing environments.

## Data Availability

The original contributions presented in the study are included in the article; further inquiries can be directed to the corresponding author.
